# Test-time local training of neural network for tabular data

**DOI:** 10.1038/s41598-025-31491-3

**Published:** 2025-12-09

**Authors:** Myeonginn Kang, Seokho Kang

**Affiliations:** Department of Industrial Engineering, Sungkyunkwan University, Jangan-gu, 16419 Suwon, Republic of Korea

**Keywords:** Engineering, Mathematics and computing

## Abstract

Generally, a neural network is globally trained using the dataset provided in the training phase to optimize its parameters. The trained neural network is then used to make predictions for query instances in the inference phase. This global learning approach leads to a neural network that performs well universally across various query instances. However, it may overlook local structures in some low-density data regions, potentially degrading generalization performance in these regions. Although several test-time adaptation methods have been explored in recent years, they are typically designed for vision domains and are not intended for or do not readily transfer to tabular data. In this study, we propose a test-time local training method, specifically tailored for tabular data, to make the neural network better reflect the local structure around the query instance during the inference phase. Given a query instance, the proposed method finds the nearest neighbors of that instance from the training dataset. It then localizes the globally trained neural network by fine-tuning with these nearest neighbors to better accommodate the local structure around the query instance. The localized neural network is finally used to make a prediction for the query instance. Through experiments conducted on tabular benchmark datasets for regression and classification tasks, we demonstrate that the proposed method significantly enhances the generalization ability of neural networks.

## Introduction

Neural networks have successfully demonstrated their effectiveness in various industrial fields, including manufacturing^1^, finance^2^, and medicine^3^. In real-world applications, a neural network is generally implemented based on the global learning approach as follows. In the training phase, given a training dataset $\mathcal {D}=\{(\textbf{x}_i, y_i)\}_{i=1}^N$ where $\textbf{x}_i$ and $y_i$ are the input vector and label of the *i*-th training instance, a global model *f* is trained globally by learning from $\mathcal {D}$ by minimizing a global objective function^4^. In the inference phase, given a query instance $\textbf{x}_*$, the model *f* is used to make a prediction for the label as $\hat{y}_* = f(\textbf{x}_*)$. This global learning approach enables a single model to effectively generalize across the underlying data distribution of the training dataset. However, there is a risk that the model may overlook local variations or nuances in low-density data regions because the global objective function tends to be biased toward high-density regions^5^.

On the other hand, the local learning approach focuses on making predictions by leveraging the local information surrounding query instances^6–8^. In the training phase, it does nothing or minor processing of the training dataset $\mathcal {D}$. In the inference phase, it builds a local model $g_*$ specialized for the given query instance $\textbf{x}_*$ based on the local neighborhood in the training dataset $\mathcal {D}$, and then, makes a prediction as $\hat{y}_* = g_*(\textbf{x}_*)$. While this approach excels at leveraging local information around the query instance for label prediction, it incurs high computational costs in the inference phase because a new local model must be trained for each query instance.

In this study, we propose a test-time local training (TTLT) method that aims to combine the advantages of both global and local learning approaches to enhance the generalization ability of neural networks on tabular data. Rather than relying solely on global data fitting, a model can further benefit from leveraging particularly informative key instances in the learning process to enhance predictive performance^9^. Motivated by this, we fine-tune the globally trained neural network using local instances near the query instance to better predict its label. In the training phase, a neural network *f* is trained globally from the training dataset $\mathcal {D}$, like the global learning approach. In the inference phase, it finds the *k* nearest neighbors of the query instance $\textbf{x}_*$ from the training dataset $\mathcal {D}$. Then, the neural network *f* is localized for the query instance $\textbf{x}_*$ by fine-tuning with these nearest neighbors. The final prediction is obtained using the localized neural network. This additional fine-tuning process for each query instance enables the globally trained neural network to adapt to the local region surrounding that query instance, thereby improving the accuracy of its prediction. We investigate the effectiveness of the proposed method by evaluating the predictive performance of neural networks on 9 regression and 9 classification benchmark datasets.

## Related work

### Local learning

Local learning methods predict the label for a query instance based on local information around that instance^8^. This is accomplished during the inference phase by prioritizing and weighing more heavily the training instances that are in closer proximity to the query instance. The most representative method is *k*-nearest neighbors (*k*-NN)^7^. Given a query instance, it identifies the *k* nearest training instances from the training dataset. The label of the query instance is then predicted by aggregating the labels of these *k* instances. Another representative method is locally weighted linear regression (LWLR)^6^. Given a query instance, this method trains a linear model by assigning larger weights to training instances closer to the query instance and smaller weights to those farther away. The linear model is utilized to make a prediction for the query instance.

Despite their simplicity and effectiveness, the performance of local learning methods is sensitive to their hyperparameter settings, with optimal settings varying across different query instances. Optimizing hyperparameters for each query instance proves challenging. Moreover, these methods necessitate longer inference times and incur higher computational costs for making predictions, particularly when dealing with large numbers of training instances and features. To address these issues, research efforts have focused on efficiently selecting the hyperparameter values^10–15^ and on improving the inference speed^16–18^.

In this study, we adapt the concept of local learning to enhance the predictive performance of a neural network globally trained during the training phase. The proposed method fine-tune the neural network in the inference phase to better capture local information surrounding the query instance.

### Model adaptation

Model adaptation refers to methods that adapt an existing model to specific query instances while leveraging knowledge within the model, with the aim of achieving more accurate predictions for a targeted task. In the context of this study, numerous previous studies related to the adaptation of neural networks have been identified, which can be broadly categorized into two main research directions.

The first research direction is transfer learning, which involves fine-tuning a pre-trained model using a dataset for a specific target task to improve predictive performance for that task^19^. The pre-trained model can be acquired through supervised learning using a large labeled training dataset for a related source task^20,21^, or through self-supervised learning without the need for label information^22–25^. Transfer learning significantly accelerates the training process for the target task by benefiting from the knowledge stored in the pre-trained model. Also, it reduces the need for an extensive labeled training dataset for the target task to achieve sufficient predictive performance.

The second research direction is test-time adaptation, which involves adapting a model to query instances before making predictions during the inference phase^26^. It primarily addresses the problem of distribution shifts, where the distribution of query instances differs from that of the training dataset. Existing studies have investigated four scenarios based on how query instances are provided in the inference phase: test-time domain adaptation, test-time batch adaptation, test-time instance adaptation, and online test-time adaptation. Test-time domain adaptation addresses the scenario where a set of query instances is accessible at once, adapting the model by fine-tuning with these instances before making predictions^27–29^. Test-time batch adaptation involves adapting the model using a mini-batch that contains a few query instances^30–32^. Similarly, test-time instance adaptation adapts the model using a single query instance^33,34^. Online test-time adaptation adapts the model in an online manner, assuming a series of mini-batches are sequentially observed, one after another^35–38^.

In relation to transfer learning, the proposed method can be viewed as fine-tuning of an already trained model using a subset of the original training dataset, which comprises the nearest neighbors of the query instance. The target task is locally specialized prediction for the query instance. In relation to test-time adaptation, the proposed method aligns with the scenario of test-time instance adaptation, adapting a model to a single query instance. It aims to address the discrepancy between the global data distribution and the local data distribution surrounding the query instance. While most existing methods for test-time adaptation are designed for image classification and do not readily transfer to tabular data, the proposed method is specifically applicable to both regression and classification tasks on tabular data.

## Proposed method

### Overview

**Fig. 1:**
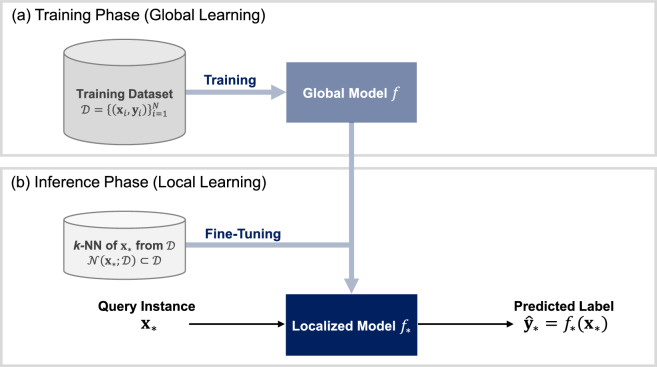
Framework of the proposed method.

In this section, we describe the proposed TTLT method for neural networks to leverage the advantages of both global and local learning approaches. The primary idea is to localize a globally trained neural network to improve predictions for specific query instances. Figure 1 schematically illustrates the overall framework of the proposed method.

The training phase of the proposed method follows the same procedure as the global learning approach. It involves constructing a neural network *f* by learning from the training dataset $\mathcal {D}=\{(\textbf{x}_i, \textbf{y}_i)\}^{N}_{i=1}$ with a global learning objective. Typically, the learning objective is defined as a loss function averaged over the training dataset, wherein the loss function measures the discrepancy between the actual and predicted labels for each training instance. This globally trained neural network itself may overlook the local structures of certain data regions, thus it is expected to result in lower predictive performance in these regions.

The inference phase involves localizing the globally trained neural network *f* through fine-tuning based on the local learning approach. Given a query instance $\textbf{x}_*$ along with the neural network *f* and the original training dataset $\mathcal {D}$, the TTLT procedure is performed to make a prediction for the query instance $\textbf{x}_*$, as follows. We first identify the set of *k*-NN instances from $\mathcal {D}$, which we denote by $\mathcal {N}(\textbf{x}_*;\mathcal {D}) \in \mathcal {D}$. We then fine-tune *f* with $\mathcal {N}(\textbf{x}_*;\mathcal {D})$ to localize it the surrounding region of $\textbf{x}_*$, resulting in a localized neural network $f_*$. The final prediction is obtained using the localized neural network $f_*$ as $\hat{\textbf{y}}_* = f_* (\textbf{x}_*)$.

### Test-time local training (TTLT)

Algorithm 1 presents the pseudocode for the TTLT procedure in making predictions for query instances in the inference phase. The details of two main steps in the procedure, nearest neighbors search and fine-tuning, are described below. Because TTLT performs adaptation at test time, the inference time and cost for each query instance mostly depend on the size of the training dataset $|\mathcal {D}|$ and the number of fine-tuning iterations *T*.

**Algorithm 1:**
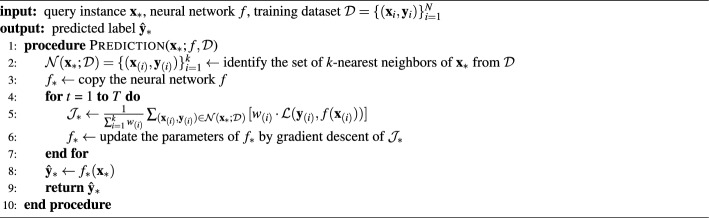
Prediction for a query instance by test-time local training (TTLT).

#### Nearest neighbors search

To identify the local information surrounding the query instance $\textbf{x}_*$, we utilize the *k*-NN instances within the training dataset $\mathcal {D}$ that are proximal to $\textbf{x}_*$. We calculate the pairwise L2 distances between the query instance $\textbf{x}_*$ and individual training instances $(\textbf{x}_i,\textbf{y}_i)$ as follows:

1 $$\begin{aligned} d_i = \Vert \textbf{x}_* - \textbf{x}_i \Vert . \end{aligned}$$

After calculating the distances for all *N* training instances, we select the *k* training instances with the smallest distances. The selected *k* instances, which we denote by $(\textbf{x}_{(1)}, \textbf{y}_{(1)}), \ldots , (\textbf{x}_{(k)}, \textbf{y}_{(k)})$, form the *k*-NN set $\mathcal {N}(\textbf{x}_*;\mathcal {D}) \subset \mathcal {D}$.

Since the *k*-NN instances are closer in distance to the query instance $\textbf{x}_*$, they are expected to share similar characteristics with $\textbf{x}_*$. We leverage these instances as local information to localize the globally trained neural network *f*.

A practical issue here is the need to access the original training dataset $\mathcal {D}$ for performing *k*-NN search for each query instance during the inference phase. This can be costly and slow down the inference speed, especially when the training dataset is large. Incorporating strategies to improve the efficiency of *k*-NN search can be considered to address this issue.

#### Fine-tuning

The globally trained neural network *f* is fine-tuned with the *k*-NN set $\mathcal {N}(\textbf{x}_*;\mathcal {D})$. The local learning objective $J_*$ is defined using $\mathcal {N}(\textbf{x}_*;\mathcal {D})$ as follows:

2 $$\begin{aligned} \mathcal {J}_* = \frac{1}{\sum _{i=1}^{k}{w_{(i)}}}\sum _{(\textbf{x}_{(i)}, \textbf{y}_{(i)}) \in \mathcal {N}(\textbf{x}_*; \mathcal {D})}{[w_{(i)} \cdot \mathcal {L}(\textbf{y}_{(i)}, f(\textbf{x}_{(i)}))]}, \end{aligned}$$

where $\mathcal {L}$ is the loss function and $w_{(i)}$ is the weight assigned to the *i*-th *k*-NN instance. The typical choice of the loss function $\mathcal {L}$ is the cross-entropy for classification tasks and the squared error for regression tasks. To assign greater weight to instances closer to the query instance $\textbf{x}_*$, we set the weight $w_{(i)}$ as the inverse of the L2 distance as:

3 $$\begin{aligned} w_{(i)} = \frac{1}{d_{(i)} + \epsilon } = \frac{1}{\Vert \textbf{x}_* - \textbf{x}_{(i)} \Vert + \epsilon }, \end{aligned}$$

where $\epsilon$ is a small positive constant to prevent the denominator from becoming zero.

For fine-tuning, we update the parameters of *f* by minimizing the objective $\mathcal {J}_*$ over *T* iterations using a small learning rate. This process enables the neural network to be localized to further reflect the local information relevant to the query instance $\textbf{x}_*$, thereby expected to make more accurate predictions in the corresponding local region. The fine-tuned version of the neural network, which we denote by $f_*$, is used to make a prediction for the query instance $\textbf{x}_*$.

## Experiments

### Benchmark datasets

We evaluated the effectiveness of the proposed method on 18 tabular benchmark datasets collected from the UCI machine learning repository^39^ and KEEL dataset repository^40^, 9 for classification and 9 for regression. Among the benchmark datasets, *Telemonitoring* and *Indoorloc* had multiple labels. For these, we selected ‘total’ and ‘longitude’, respectively.

As preprocessing, all numeric variables were standardized to have zero mean and unit variance. For each benchmark dataset, 80% of instances were used as the training dataset and the remaining 20% were reserved as the test dataset for evaluating the generalization performance.

### Experimental settings

For the proposed method, we determined the neural network architecture for each benchmark dataset to maximize the predictive performance of the globally trained neural network. The search spaces for the number of hidden layers and the number of units per hidden layer were {1, 2, 3} and {50, 100, 200 500}, respectively. The tanh activation function was applied to the hidden units. The output layer contained one linear unit for regression tasks and *c* units with softmax activation for *c*-class classification tasks. It should be noted that the best architectures differed among the benchmark datasets.

In the training phase, 25% of the training dataset was reserved as the validation set for early-stopping. We used the Adam optimizer with a learning rate of $10^{-3}$, a mini-batch size of 50, and a weight decay factor of $10^{-5}$. The learning rate was decayed by a factor of 0.1 if validation performance did not improve over 50 consecutive epochs. For early-stopping, the patience and the maximum number of epochs were set as 100 and 500, respectively.

In the inference phase, the fine-tuning was performed using the Adam optimizer with a learning rate of $10^{-5}$ and a weight decay factor of $10^{-5}$. For regression tasks, the number of nearest neighbors for *k*-NN search (*k*) and the number of training iterations for fine-tuning (*T*) were set to 100 and 100, respectively. For classification tasks, these hyperparameters were set to 10 and 100, respectively. We also investigated the effects of *k* and *T* on the predictive performance by varying their values as $\{1, 3, 5, 10, 20, 50, 100\}$.

We implemented the proposed method using the PyTorch library in Python. The source code used in this study is available at [https://github.com/mi-kang228/test_time_local_training].

### Compared methods

For regression tasks, the proposed method (NN$_\text {TTLT}$) was compared to two local learning methods and three global learning methods: *k*-NN and LWLR for local learning; linear regression (LR), random forest (RF), and globally trained neural network (NN$_\text {global}$) for global learning. For classification tasks, among the compared methods LWLR and LR were replaced with locally weighted softmax regression (LWSR) and softmax regression (SR), respectively.

The hyperparameters of the baseline methods were configured as follows. For *k*-NN, the number of nearest neighbors *k* was searched from $\{1, 3, 5, 10, 20, 30, 50, 100\}$. For LWLR and LWSR, L2 regularization with a factor of $10^{-5}$ was applied and the weight for each training instance $(\textbf{x}_i, \textbf{y}_i)$ was set as $w_i = \exp {\left( -\frac{\Vert \textbf{x}_*-\textbf{x}_i\Vert ^2}{2\sigma ^2} \right) }$, where the hyperparameter $\sigma ^2$ was searched from $\{0.01, 0.1, 1, 10, 100\}$. For LR and SR, L2 regularization with a factor of $10^{-5}$ was also applied.

We implemented all the baseline methods except NN$_\text {global}$ using the scikit-learn library in Python. The configurations unspecified above were set to the defaults in this library. NN$_\text {global}$ was implemented using PyTorch, as was the proposed method.

**Table 1:** RMSE comparison results on regression benchmark datasets (mean ± standard deviation). The best value in each row is shown in bold, and the second-best value is underlined.

Dataset	No. instances $\times$ no. features	Local learning		Global learning			Proposed
		*k*-NN	LWLR	LR	RF	NN$_\text {global}$	NN$_\text {TTLT}$
Bikesharing	17,379 $\times$ 12	0.5699 ± 0.0077	0.6313 ± 0.0108	0.7795 ± 0.0099	0.2404 ± 0.0059	0.2344 ± 0.0067	0.2242 ± 0.0072**
Compactiv	8192 $\times$ 21	0.2429 ± 0.0381	0.2072 ± 0.0348	0.5203 ± 0.0233	0.1400 ± 0.0167	0.1345 ± 0.0085	**0.1298 ± 0.0067**
Cpusmall	8192 $\times$ 12	0.1885 ± 0.0105	0.1645 ± 0.0055	0.5274 ± 0.0177	**0.1518 ± 0.0023**	0.1587 ± 0.0055	0.1553 ± 0.0050
CTscan	53,500 $\times$ 385	0.0081 ± 0.0005	0.0072 ± 0.0005**	0.3704 ± 0.0031	0.0604 ± 0.0026	0.0225 ± 0.0043	0.0172 ± 0.0050
Indoorloc	19,937 $\times$ 525	0.0562 ± 0.0080	0.2392 ± 0.0087	0.2045 ± 0.0016	0.0238 ± 0.0017**	0.0525 ± 0.0036	0.0438 ± 0.0047
Mv	40,768 $\times$ 10	0.1369 ± 0.0010	0.0362 ± 0.0006	0.4320 ± 0.0043	0.0070 ± 0.0026	0.0050±0.0003	0.0043 ± 0.0003**
Pole	14,998 $\times$ 10	0.2133 ± 0.0074	0.3303 ± 0.0070	0.7317 ± 0.0055	0.1116 ± 0.0047	0.0549 ± 0.0028	**0.0537 ± 0.0025**
Puma32h	8192 $\times$ 32	0.8878 ± 0.0155	0.8982 ± 0.0183	0.8844 ± 0.0149	0.2578 ± 0.0029	0.2567 ± 0.0037	0.2487 ± 0.0030**
Telemonitoring	5875 $\times$ 20	0.3633 ± 0.0185	0.4824 ± 0.0159	0.8703 ± 0.0164	0.0358 ± 0.0043**	0.0762 ± 0.0228	0.0708 ± 0.0232
Average rank	–	4.22±0.92	4.44 ± 1.42	5.67 ± 0.67	2.56 ± 1.26	2.56 ± 0.68	**1.56 ± 0.68**

**Table 2:** Error rate comparison results on classification benchmark datasets (mean ± standard deviation). The best value in each row is shown in bold, and the second-best value is underlined.

Dataset	No. instances $\times$ no. features (no. classes)	Local learning		Global learning			Proposed
		*k*-NN	LWSR	SR	RF	NN$_\text {global}$	NN$_\text {TTLT}$
Drybean	13,611 $\times$ 16 (7)	0.0749 ± 0.0025	0.0704 ± 0.0023	0.0771 ± 0.0029	0.0758 ± 0.0031	0.0711 ± 0.0035	**0.0683 ± 0.0029**
Letter	20,000 $\times$ 16 (26)	0.0473 ± 0.0032	0.0369 ± 0.0019	0.2255 ± 0.0044	0.0341 ± 0.0022	0.0253 ± 0.0026	0.0189 ± 0.0018**
Magic	19,020 $\times$ 10 (2)	0.1570 ± 0.0051	0.1319 ± 0.0049	0.2081 ± 0.0066	**0.1200 ± 0.0059**	0.1228 ± 0.0053	0.1247 ± 0.0042
Optdigits	5620 $\times$ 64 (10)	0.0190 ± 0.0028	0.0927 ± 0.0061	0.0414 ± 0.0042	0.0180 ± 0.0029	0.0230 ± 0.0026	0.0147 ± 0.0031*
Pageblocks	5473 $\times$ 10 (5)	0.0335 ± 0.0049	0.0300 ± 0.0053	0.0384 ± 0.0052	0.0271 ± 0.0040	0.0301 ± 0.0054	0.0260 ± 0.0045*
Penbased	10,992 $\times$ 20 (10)	0.0056 ± 0.0011	0.0057 ± 0.0011	0.0454 ± 0.0039	0.0078 ± 0.0009	0.0057 ± 0.0012	0.0041 ± 0.0008**
Satimage	6435 $\times$ 36 (6)	0.0942 ± 0.0063	0.0923 ± 0.0067	0.1437 ± 0.0042	0.0869 ± 0.0076	0.0932 ± 0.0073	0.0788 ± 0.0070*
Texture	5500 $\times$ 40 (11)	0.0111 ± 0.0039	0.0021 ± 0.0014	0.0027 ± 0.0020	0.0209 ± 0.0035	0.0020 ± 0.0017	**0.0012 ± 0.0007**
Waveform	5000 $\times$ 21 (3)	0.1480 ± 0.0094	0.1611 ± 0.0114	**0.1322 ± 0.0093**	0.1503 ± 0.0089	**0.1322 ± 0.0081**	0.1331 ± 0.0097
Average Rank	–	4.22 ± 1.03	3.83 ± 1.29	5.17 ± 1.45	3.44 ± 1.71	2.89 ± 0.97	**1.44 ± 0.83**

### Results and discussion

The predictive performance for regression tasks was evaluated in terms of root mean squared error (RMSE) calculated on the test dataset. The predictive performance for classification tasks was evaluated in terms of misclassification error rate on the test dataset. All experiments were repeated ten times independently with different random seeds. For each benchmark dataset, we report the predictive performance averaged over the ten repetitions.

Table 1 compares the predictive performance of the baseline and proposed methods on regression benchmark datasets in terms of RMSE. Table 2 provides comparison results on classification benchmark datasets in terms of error rate. To evaluate statistical significance, the t-test was conducted comparing the best and second-best methods per dataset. Single and double asterisks (* and **) indicate p-values less than 0.05 and 0.01, respectively. The results demonstrated that the proposed method improved the predictive performance of neural networks. Compared to NN$_\text {global}$, NN$_\text {TTLT}$ consistently yielded a lower RMSE or error rate for all benchmark datasets, except for $\textit{Magic}$ and $\textit{Waveform}$. On these two datasets, the performance gap was negligible. Notably, by applying the TTLT procedure, the RMSE was reduced by over 10% on three datasets out of 9 regression datasets, and the error rate was reduced by over 10% on six datasets out of 9 classification datasets. Compared to other baselines including various local and global learning methods, NN$_\text {TTLT}$ achieved the lowest average rank across the benchmark datasets. While some baseline methods exhibited the best performance on certain datasets, their superiority was inconsistent across benchmark datasets with performance deteriorating on others.

**Fig. 2:**
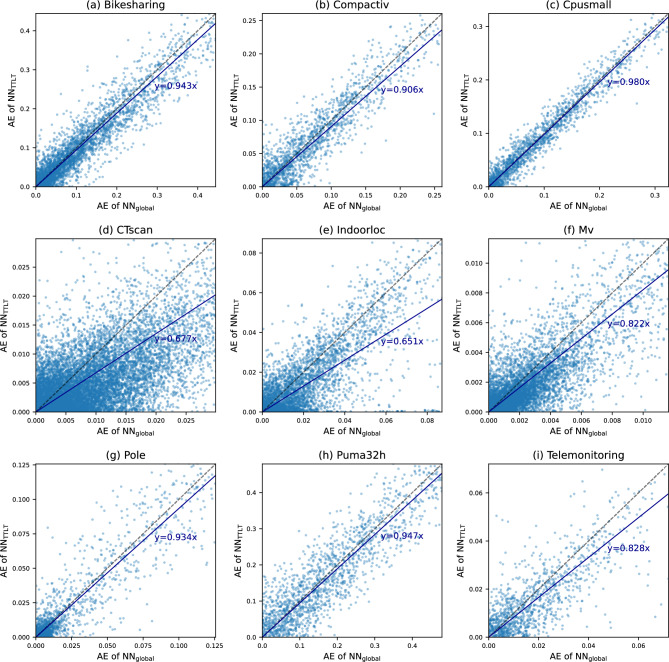
Comparison of instance-wise AE between NN$_\text {global}$ and NN$_\text {TTLT}$ on regression benchmark datasets.

**Fig. 3:**
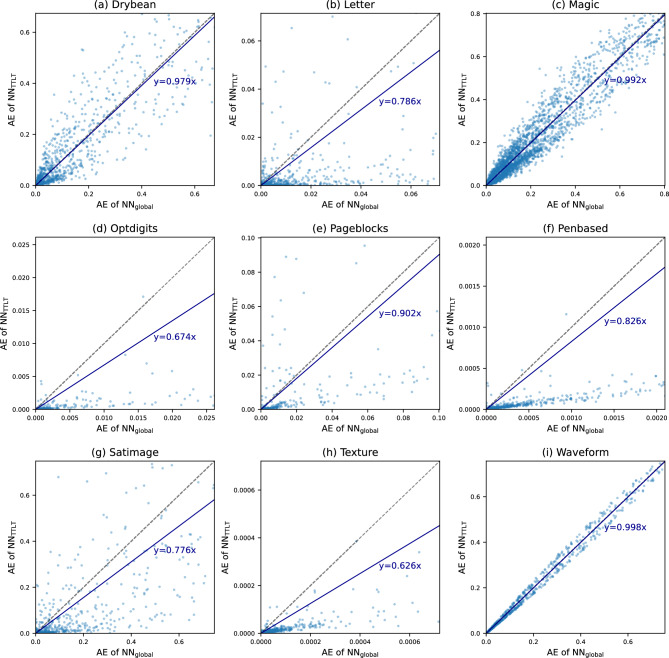
Comparison of instance-wise AE between NN$_\text {global}$ and NN$_\text {TTLT}$ on classification benchmark datasets.

We evaluated instance-level performance improvements of the proposed method compared to globally trained neural networks by plotting the instance-wise absolute error (AE) of NN$_\text {TTLT}$ against NN$_\text {global}$ for each benchmark dataset. For regression tasks, the AE is calculated as $|y_i - \hat{y}_i |$, where we denote the ground-truth and predicted label for the *i*-th test instance as $y_i\in \mathbb {R}$ and $\hat{y}_i\in \mathbb {R}$. For classification tasks, the AE is calculated as $0.5 \cdot \Vert \textbf{y}_i - \hat{\textbf{y}}_i \Vert _1$, which ranges between 0 and 1, assuming the ground-truth label $\textbf{y}_i\in \{0,1\}^c$ in the form of a *c*-dimensional one-hot vector and the predicted label $\hat{\textbf{y}}_i\in [0,1]^c$ in the form of a softmax response vector. The results for the regression and classification benchmark datasets are illustrated in Figs. 2and 3, respectively. We observed an overall tendency that NN$_\text {TTLT}$ yielded a lower AE compared to NN$_\text {global}$. Upon drawing linear trend lines, the slopes were below 1 for all benchmark datasets. The proposed method tended to be more effective for test instances for which the AE of NN$_\text {global}$ was higher. Meanwhile, the proposed method was not always effective for every test instance, as indicated by several points situated above the diagonal line in the plots.

We further conducted a sensitivity analysis to investigate how the two main hyperparameters *k* and *T* affected predictive performance. Detailed results are provided in the Information.

## Conclusion

In this study, we presented the TTLT method for predictive modeling on tabular data. This method improves a globally trained neural network for each query instance through local learning during the inference phase, aiming to enhance the accuracy of predictions for the query instance. Given a query instance, its local information is identified using its *k*-NN instances from the original training dataset. By fine-tuning with these *k*-NN instances, the neural network becomes localized to better reflect the local information surrounding the query instance. This localized neural network is then used to make the final prediction. Through experiments conducted on tabular benchmark datasets on both regression and classification, we demonstrated that the proposed method not only outperformed globally trained neural networks but also exceeded the performance of other local and global learning methods on average.

The primary advantage of the proposed method is its versatility across various real-world scenarios. By simply fine-tuning existing globally trained neural networks, it can improve the predictive performance of any supervised learning-based prediction task, including classification and regression. Moreover, it imposes no constraints on the architecture or learning objectives of the neural networks.

While the proposed method successfully reduced the prediction error on average, we identify three issues that could serve as potential directions for further research to enhance its effectiveness. First, the proposed method incurs a high computational cost during the inference phase due to its test-time nature, which increases with the size of the training dataset and the number of fine-tuning iterations. To alleviate this, improving the efficiency of *k*-NN search and updating only parts of the neural network, rather than the entire network, could be considered. Second, we observed an increase in error for some query instances compared to a globally trained neural network. The overall predictive performance could be further improved by selectively applying the TTLT procedure only when it is expected to reduce the prediction error, rather than uniformly applying it to every query instance. This approach could not only enhance prediction accuracy but also reduce computational costs. Third, the proposed method fine-tunes the globally trained network from scratch for every query instance without leveraging information from previous queries. To improve efficiency in real-world scenarios, we can instead initialize the network for each new query using the one fine-tuned to adapt to on earlier queries. Sequentially fine-tuning the network while preventing catastrophic forgetting may reduce the required number of fine-tuning iterations per query, thereby lowering the overall computational cost.

## Supplementary Information

Supplementary Information.

## Data Availability

The source code used in this study is available at [https://github.com/mi-kang228/test_time_local_training]. The datasets used in this study are available in the UCI machine learning repository [https://archive.ics.uci.edu/] and the KEEL dataset repository [https://sci2s.ugr.es/keel/].

## References

[1] Wang, J., Ma, Y., Zhang, L., Gao, R. X. & Wu, D. Deep learning for smart manufacturing: Methods and applications. *J. Manufact. Syst.* **48**, 144–156 (2018).

[2] Heaton, J. B., Polson, N. G. & Witte, J. H. Deep learning for finance: Deep portfolios. *Appl. Stochastic Models Business Industry* **33**, 3–12 (2017).

[3] Piccialli, F., Di Somma, V., Giampaolo, F., Cuomo, S. & Fortino, G. A survey on deep learning in medicine: Why, how and when?. *Inform. Fusion* **66**, 111–137 (2021).

[4] Abdolrasol, M. G. et al. Artificial neural networks based optimization techniques: A review. *Electronics* **10**, 2689 (2021).

[5] Huang, K.-Z., Yang, H., King, I. & Lyu, M. R. *Machine Learning: Modeling Data Locally and Globally* (Springer Science & Business Media, 2008).

[6] Atkeson, C. G., Moore, A. W. & Schaal, S. Locally weighted learning. *Artif. Intell. Rev.* **11**, 11–73 (1997).

[7] Fix, E. & Hodges, J. L. Discriminatory analysis. nonparametric discrimination: Consistency properties. *Int. Stat. Rev.* **57**, 238–247 (1989).

[8] Bottou, L. & Vapnik, V. Local learning algorithms. *Neural Comput.* **4**, 888–900 (1992).

[9] Zhang, Y.-X., Zhou, Z., He, X., Adhikary, A. R. & Dutta, B. Data-driven knowledge fusion for deep multi-instance learning. *IEEE Trans. Neural Netw. Learn. Syst.* **36**, 8292–8306 (2024). 10.1109/TNNLS.2024.3436944 39120987

[10] Sun, S. & Huang, R. An adaptive k-nearest neighbor algorithm. In *Proceedings of International Conference on Fuzzy Systems and Knowledge Discovery* (2010).

[11] Wang, J., Neskovic, P. & Cooper, L. N. Neighborhood size selection in the k-nearest-neighbor rule using statistical confidence. *Pattern Recognit.* **39**, 417–423 (2006).

[12] García-Pedrajas, N., Del Castillo, J. A. R. & Cerruela-García, G. A proposal for local k values for k-nearest neighbor rule. *IEEE Trans. Neural Netw. Learning Syst.* **28**, 470–475 (2015). 10.1109/TNNLS.2015.2506821 26731778

[13] Zhang, S., Cheng, D., Deng, Z., Zong, M. & Deng, X. A novel kNN algorithm with data-driven k parameter computation. *Pattern Recognit. Lett.* **109**, 44–54 (2018).

[14] Pan, Z., Wang, Y. & Pan, Y. A new locally adaptive k-nearest neighbor algorithm based on discrimination class. *Knowl.-Based Syst.* **204**, 106185 (2020).

[15] Hassanat, A.B., Abbadi, M.A., Altarawneh, G.A. & Alhasanat, A.A. Solving the problem of the k parameter in the kNN classifier using an ensemble learning approach. *Int. J. Comput. Sci. Inform. Security*. 33–39 (2014).

[16] Gallego, A. J., Rico-Juan, J. R. & Valero-Mas, J. J. Efficient k-nearest neighbor search based on clustering and adaptive k values. *Pattern Recognit.* **122**, 108356 (2022).

[17] Wang, Y. & Wang, Z.-O. A fast kNN algorithm for text categorization. In *Proceedings of International Conference on Machine Learning and Cybernetics* (2007).

[18] Cecchin, L., Baumgärtner, K., Gering, S. & Diehl, M. Locally weighted regression with approximate derivatives for data-based optimization. In *Proceedings of European Control Conference* (IEEE, 2023).

[19] Zhuang, F. et al. A comprehensive survey on transfer learning. *Proc. IEEE* **109**, 43–76 (2020).

[20] Deng, J. et al. ImageNet: A large-scale hierarchical image database. In *Proceedings of IEEE/CVF Conference on Computer Vision and Pattern Recognition*, 248–255 (2009).

[21] Lin, T.-Y. et al. Microsoft COCO: Common objects in context. In *Proceedings of European Conference on Computer Vision*, 740–755 (2014).

[22] Chen, T., Kornblith, S., Norouzi, M. & Hinton, G. A simple framework for contrastive learning of visual representations. In *Proceedings of International Conference on Machine Learning*, 1597–1607 (2020).

[23] He, K., Fan, H., Wu, Y., Xie, S. & Girshick, R. Momentum contrast for unsupervised visual representation learning. In *Proceedings of IEEE/CVF Conference on Computer Vision and Pattern Recognition*, 9729–9738 (2020).

[24] Grill, J.-B. et al. Bootstrap your own latent-A new approach to self-supervised learning. In *Adv. Neural Inform. Process. Syst*. 21271–21284 (2020).

[25] He, K. et al. Masked autoencoders are scalable vision learners. In *Proceedings of IEEE/CVF Conference on Computer Vision and Pattern Recognition*, 16000–16009 (2022).

[26] Liang, J., He, R. & Tan, T. A comprehensive survey on test-time adaptation under distribution shifts. arXiv preprint arXiv:2303.15361 (2023).

[27] Liang, J., Hu, D. & Feng, J. Do we really need to access the source data? Source hypothesis transfer for unsupervised domain adaptation. In *Proceedings of International Conference on Machine Learning*, 6028–6039 (2020).

[28] Kundu, J. N., Venkat, N. & Babu, R.V. et al. Universal source-free domain adaptation. In *Proceedings of IEEE/CVF Conference on Computer Vision and Pattern Recognition*, 4544–4553 (2020).

[29] Li, R., Jiao, Q., Cao, W., Wong, H.-S. & Wu, S. Model adaptation: Unsupervised domain adaptation without source data. In *Proceedings of IEEE/CVF Conference on Computer Vision and Pattern Recognition*, 9641–9650 (2020).

[30] Schneider, S. et al. Improving robustness against common corruptions by covariate shift adaptation. In *Advances in Neural Information Processing Systems* (2020).

[31] Yuan, L., Xie, B. & Li, S. Robust test-time adaptation in dynamic scenarios. In *Proceedings of IEEE/CVF Conference on Computer Vision and Pattern Recognition*, 15922-15932 (2023).

[32] Nguyen, A. T., Nguyen-Tang, T., Lim, S.-N. & Torr, P. H. Tipi: Test time adaptation with transformation invariance. In *Proceedings of IEEE/CVF Conference on Computer Vision and Pattern Recognition*, 24162–24171 (2023).

[33] Zhang, M., Levine, S. & Finn, C. MEMO: Test time robustness via adaptation and augmentation. In *Advances in Neural Information Processing Systems* (2022).

[34] Wang, Q., Fink, O., Van Gool, L. & Dai, D. Continual test-time domain adaptation. In *Proceedings of IEEE/CVF Conference on Computer Vision and Pattern Recognition*, 7201–7211 (2022).

[35] Gong, T. et al. NOTE: Robust continual test-time adaptation against temporal correlation. In *Advances in Neural Information Processing Systems* (2022).

[36] Niu, S. et al. Efficient test-time model adaptation without forgetting. In *Proceedings of International Conference on Machine Learning* (2022).

[37] Wang, D., Shelhamer, E., Liu, S., Olshausen, B. & Darrell, T. Tent: Fully test-time adaptation by entropy minimization. In *Proceedings of International Conference on Learning Representations* (2021).

[38] Chen, D., Wang, D., Darrell, T. & Ebrahimi, S. Contrastive test-time adaptation. In *Proceedings of IEEE/CVF Conference on Computer Vision and Pattern Recognition*, 295–305 (2022).

[39] Kelly, M., Longjohn, R. & Nottingham, K. The UCI machine learning repository (2023).

[40] Alcalá-Fdez, J. et al. KEEL data-mining software tool: Data set repository, integration of algorithms and experimental analysis framework. *J. Multiple-Valued Logic Soft Comput.* **17**, 255–287 (2011).

