# Supplementary material for: Test-time local training of neural network for tabular data
Source: Sci Rep. 2025 Dec 9;16:1863. doi: 10.1038/s41598-025-31491-3 (PMC12804743; doi:10.1038/s41598-025-31491-3)
Supplement: Supplementary file 1 — Supplementary Information. [file 41598_2025_31491_MOESM1_ESM.pdf]

Supplementary Material

Test-Time Local Training of Neural Network for Tabular Data

Myeonginn Kang and Seokho Kang*

Department of Industrial Engineering, Sungkyunkwan University, Jangan-gu, Suwon 16419, Republic of Korea

*email: s.kang@skku.edu

Hyperparameter Sensitivity Analysis

To investigate how the two main hyperparameters k and T affected predictive performance, we conducted a sensitivity analysis by varying their values as $\{0, 1, 3, 5, 10, 20, 50, 100\}$. It should be noted that setting k and T to 0 is equivalent to $\text{NN}_{\text{global}}$. The results for the regression and classification benchmark datasets are shown in Figs. S1 and S2, respectively. We observed a general tendency where increasing k and T led to lower RMSE or error rates in most cases, at the expense of higher computational costs. However, in some benchmark datasets, increasing k and T beyond a certain level resulted in a deterioration of predictive performance. Therefore, it is important to choose these hyperparameters appropriately based on the specific context and the trade-offs involved.

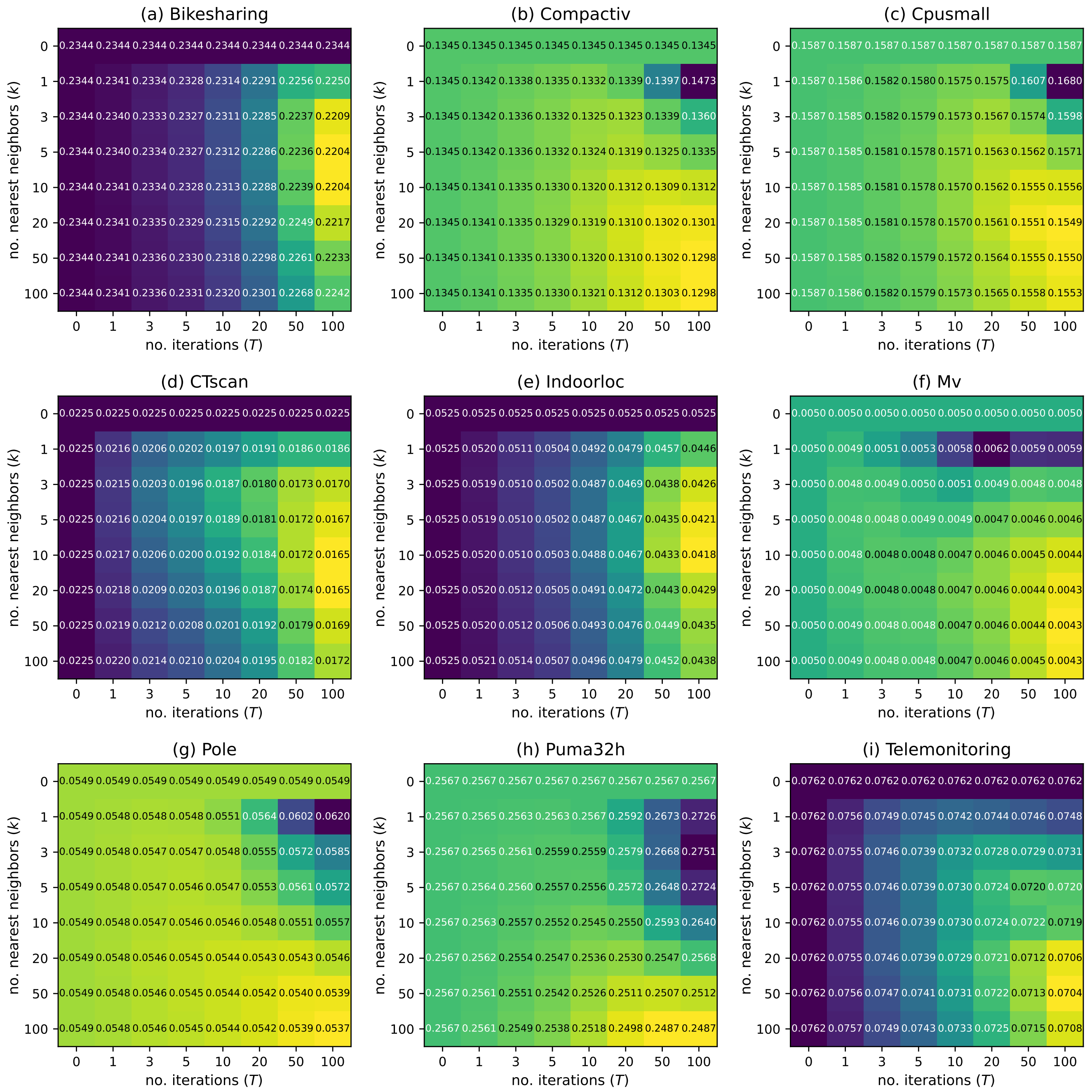

Figure S1. The effects of hyperparameters k and T on the proposed method for regression benchmark datasets (in RMSE)

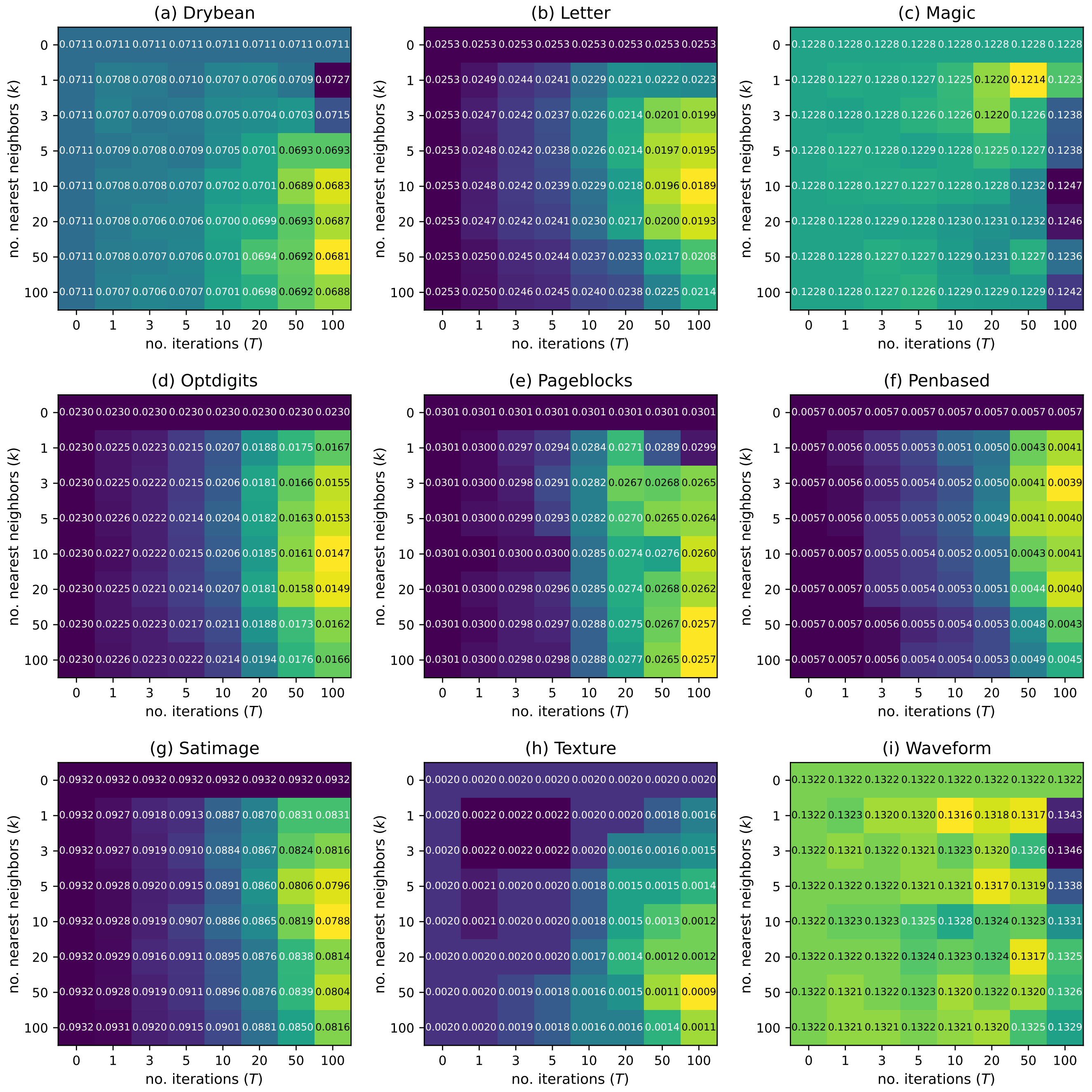

Figure S2. The effects of hyperparameters k and T on the proposed method for classification benchmark datasets (in error rate)
